# The crosstalk between mitochondrial quality control and metal-dependent cell death

**DOI:** 10.1038/s41419-024-06691-w

**Published:** 2024-04-27

**Authors:** Qi-yuan Zhou, Chao Ren, Jing-yan Li, Lu Wang, Yu Duan, Ren-qi Yao, Ying-ping Tian, Yong-ming Yao

**Affiliations:** 1 Department of Emergency, the Second Hospital of Hebei Medical University, Shijiazhuang, 050000 China; 2 Department of Pulmonary and Critical Care Medicine, Beijing Chaoyang Hospital, Capital Medical University, Beijing, 100020 China; 3 Department of Critical Care Medicine, the First Medical Center of Chinese PLA General Hospital, Beijing, 100853 China; 4 Department of Critical Care Medicine, Affiliated Chenzhou Hospital (the First People’s Hospital of Chenzhou), Southern Medical University, Chenzhou, 423000 China; 5 Department of General Surgery, the First Medical Center of Chinese PLA General Hospital, Beijing, 100853 China; 6 Medical Innovation Research Division, Translational Medicine Research Center and the Fourth Medical Center of Chinese PLA General Hospital, Beijing, 100853 China

**Keywords:** Cell death, Autophagy, Apoptosis

## Abstract

Mitochondria are the centers of energy and material metabolism, and they also serve as the storage and dispatch hubs of metal ions. Damage to mitochondrial structure and function can cause abnormal levels and distribution of metal ions, leading to cell dysfunction and even death. For a long time, mitochondrial quality control pathways such as mitochondrial dynamics and mitophagy have been considered to inhibit metal-induced cell death. However, with the discovery of new metal-dependent cell death including ferroptosis and cuproptosis, increasing evidence shows that there is a complex relationship between mitochondrial quality control and metal-dependent cell death. This article reviews the latest research results and mechanisms of crosstalk between mitochondrial quality control and metal-dependent cell death in recent years, as well as their involvement in neurodegenerative diseases, tumors and other diseases, in order to provide new ideas for the research and treatment of related diseases.

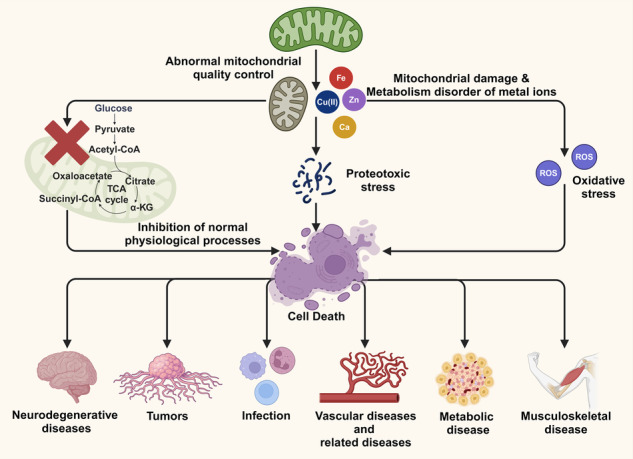

## Facts

- Mitochondrial quality control is a double-edged sword for cell death.
- Metal ions not only initiate cell death by inducing oxidative stress, but also affect the regulation of cell death.
- Mitophagy inhibits cell death via attenuation of oxidative stress, but it may also promote cell death by releasing metal ions.
- Mitochondrial quality control has a potential effect on cuproptosis.

## Questions

- Will the ROS produced by damaged mitochondria isolated from mitophagosomes affect mitophagosomes and other organelles?
- How different metal ions interfere with cell death induced by other metal ions?
- How do mitophagy-caused metal ion release and mitochondrial clearance affect cuproptosis?

## Introduction

Cell death is currently described on the basis of three modalities associated with different morphological characteristics: apoptosis, autophagy, and necrosis [1, 2]. Ferroptosis is a recently discovered form of regulated cell death (RCD) and is described as nonapoptotic, caspase-independent cell death mod accompanied by glutathione depletion [3–6]. It is mediated by iron overload, which results in reactive oxygen species (ROS) accumulation, glutathione depletion, lipid peroxidation, and ultimately cell death [7]. Metal ions are essential nutrients for host homeostasis and are involved in many physiological processes, while excessive or insufficient metal ions may lead to cell dysfunction and death [8]. Cuproptosis is caused by the binding of copper ions to lipoylated components of the tricarboxylic acid cycle, which in turn results in protein toxicity [9]. Both ferroptosis and cuproptosis show close connections to mitochondria: cuproptosis depends on lipoylated mitochondrial enzymes and the loss of Fe-S clusters [8]; the main characteristic of ferroptosis is lipid peroxidation, which is a result of excessive ROS production [10]. Mitochondria are metabolic centers of cellular energy, providing sufficient energy for cells through a series of metabolic mechanisms, including the tricarboxylic acid (TCA) cycle and oxidative phosphorylation (OXPHOS) [11]. Mitochondria plays pivotal roles in physiological functions such as fatty acid synthesis and signal processing, which further affect the fate of cells [12]. Some metal ions have been demonstrated to participate in the formation of proteins and cofactors related to mitochondrial function, and mitochondria themselves are storage centers for various metal ions in cell, which makes a close relationship between mitochondria and metal ions: the normal function of mitochondria depends on sufficient metal ion supply, and the abnormality of mitochondrial structure and function will lead to the abnormality of metal ion level and distribution [13].

Mitochondrial quality is critically involved in metal ion-dependent cell death. Multiple pathways for mitochondrial quality control regulate mitochondrial biogenesis to meet cellular metabolic, energy, and material needs through mitochondrial dynamics, mitophagy, and proteasome-mediated degradation to clear damaged or excessive mitochondria [14]. Experimental data indicated that mitophagy and ferroptosis are simultaneous processes, with mitophagy often accompanied by alterations in the expression of mitochondrial fission and fusion-related proteins such as dynamin-related protein 1 (DRP1) and mitofusin (MFN) 1/2 [15]. Moreover, the experimental results proved that mitophagy could protect cells from ferroptosis [16]. Nevertheless, increasing evidence suggests that the relationship of mitochondrial quality control with metal ion-dependent cell death is not simply inhibitory but multifactorial, involving complex interactions in the pathogenesis of various diseases [17].

## Mitochondrial damage and the maintenance of mitochondrial homeostasis

Mitochondria composed of lipid bilayer membranes efficiently provide energy for utilization by eukaryotic cells [18, 19]. Metabolite intermediates or other specific macromolecules leverage porins and outer membrane transporters (TOM) to cross the mitochondrial membrane into the inner mitochondrial space (IMS) or mitochondrial matrix, regulating the TCA cycle and oxidative respiratory chain [20]. The proton motive force established by protons entering the IMS and the electron transfer mediated by OXPHOS constitute the electrochemical gradient needed for subsequent metabolic regulation, calcium buffering, and other physiological processes [21]. Mitochondrial DNA (mtDNA) and ribosomes are critically involved in synthesizing and processing peptides and proteins. Although these mtDNA-encoded products account for only a small fraction of all mitochondrial proteins, they participate in the electron transport chain (ETC) and are crucial for OXPHOS [22].

### ROS: the primary mediator of mitochondrial damage

The cell nucleus regulates mitochondrial function via anterograde signaling, which regulates the expression of OXPHOS-related genes and activation of PPARγ coactivator 1α (PGC-1α) [23, 24]. Other organelles can also be subjected to retrograde regulation [25]. When mammalian cells acquire ATP synthesis abnormalities, mitochondria initiate a high-energy stress response, stimulating the AMPK/PGC-1α pathway and undergoing changed mitochondrial biogenesis [26]. Compared to the two aforementioned regulatory pathways, ROS are often considered biomarkers of mitochondrial damage, and ROS only at relatively low levels induce retrograde signaling [23]. Bidirectional communication to and from mitochondria ensures proper signal transduction, which is beneficial for maintaining calcium homeostasis and protein biogenesis [27].

Damaged mitochondria may become an unnecessary energy burden for the cell [28]. ROS are common inducers of mitochondrial damage [10]. Mitochondria can increase the inner membrane DHA/EPA ratio to enhance the electron transfer rate and NAD^+^/NADH ratio, thereby reducing electron leakage and ROS formation [29, 30]. Yeasts degrade ROS by expressing genes such as CTT1 and CTA1 [31, 32]. Under physiological conditions, low levels of ROS generated by mitochondria can reversibly post-translationally modify specific targets through oxidation, as a signal to regulate the body’s metabolic process. For example, when the amount of mtROS produced by mitochondria changes due to hypoxia, mtROS enhances anaerobic respiration by stabilizing HIF-1α and up-regulating key enzymes of glycolysis such as lactate dehydrogenase, reduces the dependence of cells on OXPHOS during hypoxia, and reduces the further production of mtROS. In addition, mtROS can also directly act on some proteins on the mitochondrial matrix or mitochondrial membrane, and regulate the activity of mitochondrial complex I, complex III, and complex IV through Toll-like receptors, retinoic acid-inducible gene I-like receptors and other signaling pathways, thereby affecting OXPHOS. When the ROS generation rate exceeds the clearance rate, excessive oxidation of ROS might directly damage mitochondrial lipid membranes, proteins, and mtDNA, contributing to mitochondrial dysfunction. In addition, ultraviolet light, ionizing radiation, and drug stimulation can damage mitochondria. As damaged organelles accumulate, subsequent mitochondrial failure may result in cell death [10].

### Mitochondrial biogenesis and protein quality control

**Fig. 1:**
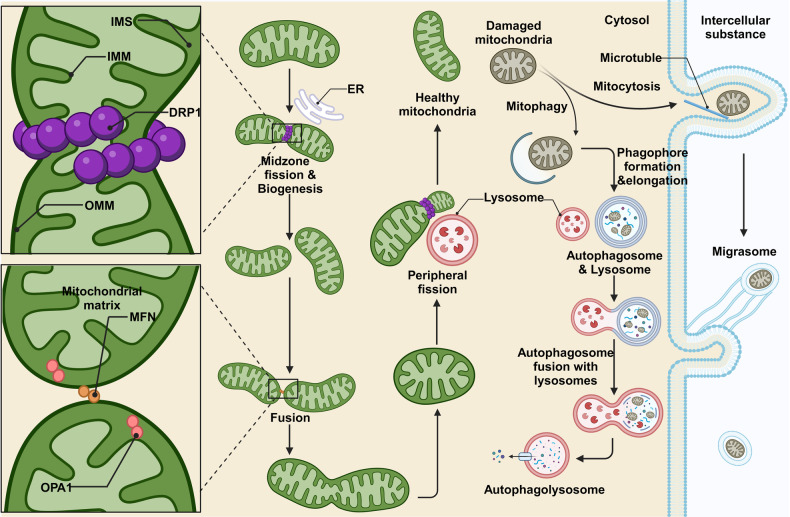
Mitochondrial dynamics. Mitochondrial midzone fission is carried out under the action of DRP1 and MFF, new mitochondria are generated by biogenesis. The ER and actin synergistically determine the division site. Some mitochondria rely on MFN and OPA1 to realize the connection and fusion of bilayer membranes to meet the metabolic needs in special cases. Mitochondria are separated by peripheral fission under the action of DRP1 and lysosomes. The damaged mitochondria are transported out of the cell by mitocytosis, or degraded by lysosomes in mitophagy. The graph was created with BioRender.com. DRP1 dynamin-related protein 1, ER endoplasmic reticulum, IMM inner mitochondrial membrane, IMS intermembrane space, MFN mitofusins, OMM outer mitochondrial membrane, OPA1 optic atrophy protein 1.

Mitochondrial quantity and quality regulation are achieved through mitochondrial biogenesis, mitochondrial dynamics, the degradation of misfolded proteins or damaged mitochondria through mechanism that involve including fission, fusion, and mitophagy (Fig. 1) [11, 33, 34].

Mitochondrial fission, including midzone and peripheral fission, is regulated by bacteria-derived DRPs, which assemble to hydrolyze GTP and thus affect mitochondrial membrane contents [35, 36]. Midzone fission is coordinated with mitochondrial biogenesis and contributes to the formation of new mitochondria to meet the needs of basal metabolism and cell proliferation rates. The ER determines the division site through a synergistic action with actin and then recruits mitochondrial fission factors (MFFs) and DRP1 to the cytoplasm [37]. As GTP is hydrolyzed, mitochondria are broken at the division site, forming two new daughter mitochondria, which are sites for subsequent protein assembly [21]. When alterations in nutritional status or mitochondrial dysfunction induce changes in the NAD^+^/NADH and AMP/ATP ratios, the expression of the PGC-1 coactivator family of proteins increases, and these proteins interact with nuclear respiratory factor (NRF)1 and 2, thereby activating the phosphorylation of AMPK and upregulating mitochondrial gene expression, transcription and replication [38, 39]. Notably, translation factors such as Pet309, which mediates the translation of cytochrome c oxidase subunit (COX)1, play important roles in the translation process, participating in the synthesis and assembly of related proteins [40]. These proteins then are localized to corresponding parts of mitochondria via membrane proteins and gradually increase the function of newly formed mitochondria [41].

mtDNA is highly susceptible to ROS-induced damage and mutations due to the lack of protection from histones and nuclear envelope [42]. The threshold effect ensures that mtDNA mutations do not have immediate results. Protein translation dysregulation or ROS overproduction may cause proteins in mitochondria to b misfolded, hindering the formation of respiratory complexes and leading to further abnormalities in tissue and organ function. Mitochondria exhibit a series of functions to prevent damaged or misfolded proteins from entering the cytoplasm and for clearing them; these functions are mediated by the mitochondrial protein translocation-associated degradation (mitoTAD) pathway, mitochondrial-associated degradation (MAD) system, mitochondrial ubiquitin‒proteasome system (UPS), mitochondrial unfolded protein response (UPR^mt^), and mitochondrial-derived vesicle carriers (MDVs).

**Fig. 2:**
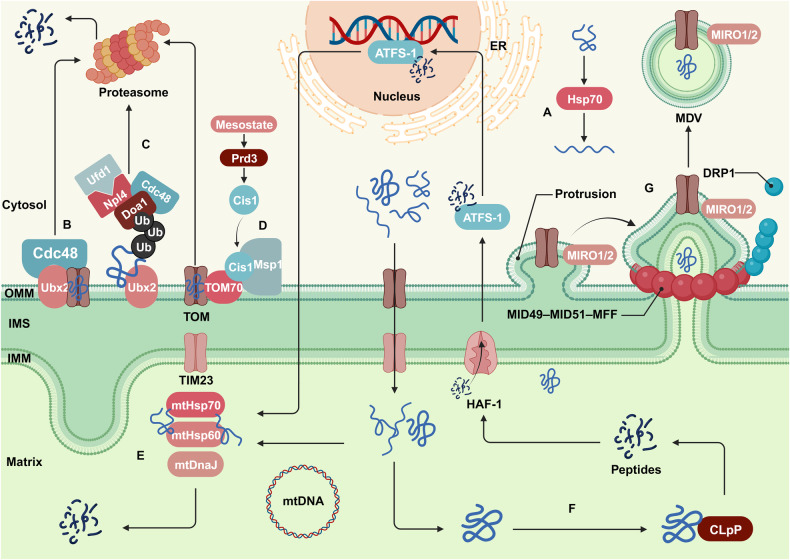
Mitochondrial protein quality control. **A** The HSP family assists the amino acid chain to fold into the correct three-dimensional structure. **B** The core protein Cdc48 of MitoTAD and its adapter Ubx2 continuously monitor the TOM channel and remove the precursor proteins that block the channel. **C** In the MAD signaling pathway, the Doa1-Cdc48^-Ufd1-Npl4^ complex and Ubx2 recognize and remove ubiquitinated proteins on the mitochondrial surface. **D** Pdr3 mediates mitoCPR, and recognizes and transfers mitochondrial protein precursors on the mitochondrial surface to proteasome degradation. **E** UPR^mt^ maintains protein homeostasis in the mitochondrial matrix. **F** Matrix protease ClpP degrades redundant misfolded proteins, and the product is transported into the nucleus to stimulate the transcription of the chaperone gene again. **G** Mitochondrial membranes encapsulate some proteins to form MDVs, which are transported to peroxisomes or lysosomes for degradation. The graph was created with BioRender.com. ATFS-1 activating transcription factor-1, Cdc48 cell division control protein 48, ClpP ATP-dependent Clp protease proteolytic subunit, HSF-1 heat shock factor 1, HSPs heat shock proteins, MDV mitochondrial-derived vesicle, MFF mitochondrial fission factors, MID49/51 mitochondrial dynamic protein 49/51, MIRO1/2 microtubule-associated motor protein 1/2, Npl4 nuclear protein localization protein 4, TIM23 mitochondrial import inner membrane translocase subunit, TOM outer membrane transporter, Ubx2 ubiquitin regulatory X domain-containing 2, Ufd1 ubiquitin-fusion degradation protein 1.

Heat shock proteins (HSPs) are highly conserved among species and facilitate in folding amino acid chains into correct three-dimensional structures and clearing misfolded amino acid chains under stress conditions (Fig. 2A). Cytoplasmic mitochondrial precursor proteins are protected and unfolded by chaperone proteins such as Hsp90 in the cytoplasm before entering mitochondria. When a precursor protein fold or import process is defective, the proteins may accumulate on the OMM or on the TOM complex, affecting normal protein entry into mitochondria and resulting in cell stress as well as damage [43]. The mitochondrial damaged protein import response (mitoCPR), mitoTAD, and MAD are essential for clearing these precursor proteins. The processes involve endoplasmic reticulum-associated degradation (ERAD) and are closely related to ubiquitination and proteasomal degradation functions of the UPS pathway [44–46]. Under mitochondrial stress conditions, mesosomes cannot enter mitochondria, and abnormal proteins accumulate and bind to the transcription factor Pdr3, triggering Pdr3-mediated mitoCPR (Fig. 2D). Pdr3 induces the expression of the OMM Cis1 protein, which binds to the translocase TOM70 and recruits AAA-ATPase Msp1 to the translocase; thus, mitochondrial protein precursors recognized on the mitochondrial surface and transferred for proteasomal degradation [47]. MitoTAD clears precursor proteins blocked in the TOM channel, and it depends on the key molecules involved in ERAD and Cdc48 (also known as AAA-ATPase p97) (Fig. 2B). Ubx2 is the adapter of Cdc48 in this process, and it specifically binding to the TOM complex under nonstress conditions and continuously monitors the TOM channel. When channels are blocked, the UBA and UBX domains of Ubx2 specifically bind to Cdc48 and introduce it to the TOM complex to capture and remove blocking precursor proteins [48]. Similarly, Cdc48 plays a critical role in the MAD pathway, forming a functional complex with Doa1, Ufd1, and Npl4 and participating in the ubiquitination and proteasomal degradation of proteins accumulated on the mitochondrial surface (Fig. 2C). After being ubiquitinated by E3 ubiquitin ligase, mitochondrial surface proteins are recognized by Doa1 via facilitation by Ubx2 and are subsequently recruited to an Ufd1-Npl4 heterodimer [49]. The Doa1-Cdc48^-Ufd1-Npl4^ complex formed by the Ufd1-Npl4 heterodimer and Cdc48 strips ubiquitinated proteins from the mitochondrial surface via the AAA-ATPase function of Cdc48 and delivers them to the proteasome for degradation [50].

As mentioned above, abnormally accumulated proteins on the mitochondrial surface and in the TOM channel are eliminated, while proteins within the matrix are processed via the UPR^mt^ pathway (Fig. 2E). Molecular chaperones such as mtHsp60, mtHsp70, and mtDnaJ are the main proteins involved in the UPR^mt^ [51]. Misfolded proteins increate the expression rats of related genes, thereby generating mitochondrial chaperones to prevent aggregation. Aberrant proteins exceeding the processing ability of mitochondrial chaperones are degraded into peptides by the matrix protease ClpP, transported to the IMS via heat shock factor-1, and then enter the nucleus through the action ATFS-1, thereafter stimulating the transcription of chaperone genes to prevent excessive aggregation of misfolded proteins (Fig. 2F) [52]. Moreover, mitochondria can eliminate damaged or potentially damaged lipids, proteins, and other cargoes through MDVs (Fig. 2G). MDVs are vesicular structures generated by mitochondrial membranes that mediate the transport of materials between mitochondria and other organelles under physiological conditions [53]. MDV synthesis is significantly increased after peroxide stimulation to provide early protective impacts under oxidative stress conditions [54]. Microtubule-associated motor proteins MIRO1/2 initiate this process, inducing the protrusion of specific regions of the mitochondrial membrane. Although DRP1 was once believed not to be involved in MDV synthesis [55], recent studies have indicated that the DRP1 receptors MID49, 51, and MFF recruit DRP1 to membrane protrusions, resulting in membrane fission and the formation of MDVs containing specific components [56]. MDVs are transported to peroxisomes via the outer membrane mitochondrial anchoring protein ligase (MAPL) or are trafficked to lysosomes via the Parkin pathway, binding to lysosomes that degrade their cargoes [53].

### Regulatory pathways for damaged mitochondria

Clearance and prevention of abnormal protein aggregation or damaged protein accumulation create the first barrier of mitochondrial quality control. Mitochondrial fusion is a “temporary compromise” to maintain a relatively stable functional state (Fig. 1). Due to the mitochondrial double-membrane structure, mitochondrial fusion requires sequential steps involving the OMM and IMM. In mammals, these steps are mediated by MFN on the OMM and optic atrophy protein 1 (OPA1) on the IMM [57]. Mitochondria undergoing fusion require MFN at contact sites on both sides of the membrane [58]. MFN1/2 anchored to the OMM mediates membrane destabilization by hydrolyzing GTP dimer at the G domain and merges the IMM with the outer membrane [59–61]. After OMM-IMM fusion, a short isomer of OPA1 (S-OPA1) induces local bending of the IMM to form tubular membranes [62]. Moreover, OPA1 forms connections and aggregates through the G domain, similar to MFN, or unique interfaces through the BSE domain, promoting the fusion and protrusion of IMM to form new mitochondrial cristae [63].

After mitochondrial fusion, the original mitochondrial contents are integrated, promoting intermitochondrial information exchange and functional and structural complementation, diluting damaged components, and maintaining mitochondrial damage and stress at low levels to ensure normal OXPHOS [11]. When damage exceeds the threshold that can be mitigated via fusion, the membrane potential decreases, concentrating damaged components at one end of a mitochondrion and separating them from the main body through peripheral fission. Peripheral fission requires DRP1 but not ER, actin, or MFF. Although the specific mechanism remains unclear, lysosomes play a regulatory role in peripheral fission without directly degrading mitochondria. FIS1-recruited TBC1D15 drives Rab7 GTP hydrolysis, promoting untethering of mitochondria and lysosomes [64] and leading to DRP1-mediated separation of the peripheral mitochondrial regions [36]. In some cases, mitochondria undergo hyperfusion induced by L-OPA1, thereby forming highly interconnected mitochondrial networks and inhibiting mitochondrial fission and mitophagy. This process generates short-term damage buffering and contributes to the maintenance of mitochondrial homeostasis [65].

Although mitochondria rely on these methods to mitigate damage, the effects are limited. When mitochondrial damage excessively accumulates and the organelle is irreversibly damaged, mitophagy is initiated to remove harmful substances and the entire damaged mitochondrion through the lysosomal pathway thereby maintaining organelle health to ensure that the overall state of the cell is not negatively affected. In contrast to MDVs, mitophagy exhibits a strong dependency on mitochondrial fission and is often triggered in the late stage of oxidative stress [54]. PTEN-induced putative kinase 1 (PINK1), a key protein in mitophagy, is processed in healthy mitochondria by TOM and TIM23 on the IMM. When the mitochondrial membrane potential is changed due to damage, PINK1 blocked from entering mitochondria accumulates on the OMM and is activated by self-phosphorylation, indirectly activating DRP1 to facilitate mitochondrial fission [66–68].

After autophagy initiation, PI3K-mediated ER-associated Ω structures gradually form double-membrane phagophores. Parkin links phosphorylated ubiquitin to the surface of highly damaged mitochondria separated via peripheral fission. The autophagy cargo receptors recognize and bind to ubiquitin on these structures and interact with LC3B through their own LIR domain on the opposite end to mediate the elongation of the phagophore membrane, thereby entrapping the connected mitochondria in the mitophagosomes being formd [69, 70]. With the involvement of SNARE proteins, mitophagosomes combine with lysosomes to form autolysosomes, ultimately degrading damaged mitochondria. To ensure mitophagy execution, signaling pathways in addition to the ubiquitination and PINK1-PRKN pathways, are involved. OMM proteins such as NIX, FUNDC1, and BNIP3 can act as mitophagy-related receptors, relying on their LIR domain to bind with LC3, thereby directing dysfunctional mitochondria to autophagosomes for degradation via receptor-mediated mitophagy [71].

The mechanisms underlying mitochondrial quality control do not function independently but depend on interplay with each other. For example, Ubx2 and Msp1 undergo functional interactions [48]. UPR^mt^ can upregulate the expression of Sesn2 to increase the mitophagy rate [72]. These linked molecules form a tightly knit defense network that helps to limit mitochondrial damage. However, our current understanding of mitochondrial quality control is in nascent stages. In 2021, Yu et al. discovered a new mitochondrial quality control mode called mitocytosis, in which abnormal mitochondria are released into the extracellular space through migrasomes [73]. Contractile fibers are left behind during cell migration, and membrane-bound structures at the fiber tips or branching points form migrasomes. Damaged mitochondria are transported to the cell periphery under the influence of the KIF5B protein, entering migrasomes and eventually detaching from the cell along with the moving migrasomes [74]. These discoveries offer new perspectives for the study of mitochondrial quality control, and other currently unknown mitochondrial quality control pathways need to be discovered and characterized.

## Mitochondria and iron

### Mitochondria and iron metabolism

**Fig. 3:**
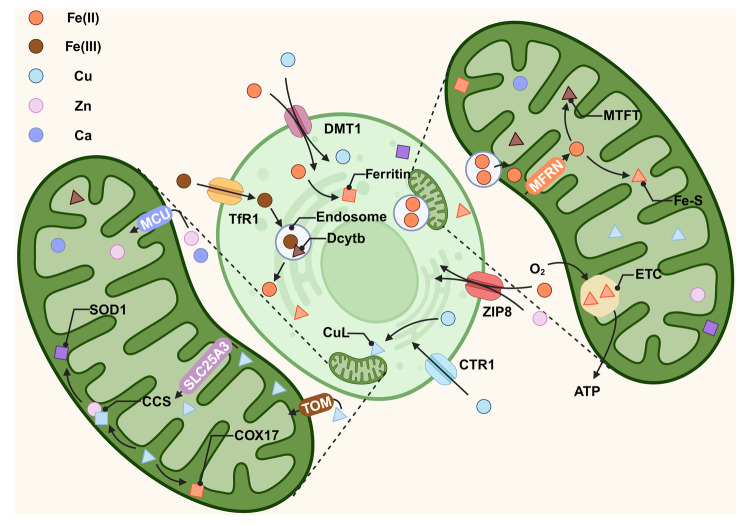
Common metal ion metabolism. Fe^3+^ is transported into the cell by TfR1 and reduces to Fe^2+^ via Dcybt. Extracellular Fe^2+^ is transported into cells by DMT1 or ZIP8, then transfers to mitochondria as ferritin or with endosomes. Iron in IMS enters the mitochondrial matrix through MFRN and is processed into the Fe-S cluster or MTFT to participate in physiological activities such as energy metabolism. Extracellular copper enters cells through DMT1 or CTR1, then processes into CuL and enters mitochondria via TOM to exert related physiological functions. Zinc and calcium in the cytoplasm are transported into mitochondria through MCU, and the former and copper are involved in synthesizing key enzymes such as SOD1. The graph was created with BioRender.com. ATP adenosine triphosphate, CCS copper chaperone for superoxide dismutase, COX17 cytochrome c oxidase subunit 17/cytochrome c oxidase copper chaperone, CTR1 copper transporter 1, Dcytb duodenal cytochrome b, DMT1 divalent metal transporter 1, ETC electron transport chain, MCU Ca^2+^ uniporter, MFRN mitoferrins, MTFT mitochondrial ferritin, SLC25A3 solute carrier family 25 member 3, SOD1 superoxide dismutase [Cu-Zn], TfR1 transferrin receptor 1, ZIP8 zinc transporter 8.

Food-derived trivalent iron is reduced to divalent iron in the duodenum and then transported into intestinal epithelial cells by the divalent metal transporter 1 (DMT1) [75, 76]. Free iron in cells is released through the basolateral membrane via ferroportin and is oxidized into trivalent iron by iron oxidases such as hephaestin, after which it is transported and utilized by transferrin (Fig. 3). Iron exists in almost all cells in the form of stable ferritin and labile iron pools (LIPs), serving as a cofactor or substrate for various proteins involved in critical biological functions, including DNA replication and lipid synthesis.

Mitochondria are crucial in iron utilization. Iron in the cytoplasm is transported into mitochondria through endosomes and mitoferrins, crossing the OMM and IMM and subsequently serving as a substrate for synthesizing different iron-containing proteins [77–79]. One of the main pathways for mitochondrial iron utilization is the Fe-S cluster pathway. Fe-S cluster proteins are widely involved in multiple cellular processes, making their synthesis and transport highly conserved across cells [78]. Iron and sulfur provided via LYR motif-containing protein 4 (LYRM4) form a [2Fe-2S] cluster facilitated by iron-sulfur cluster assembly enzyme (ISCU) serving as a scaffold protein. The [2Fe-2S] cluster is released from the core ISC complex on the ISCU dimer under the action of chaperone protein HSPA9, transferred to the glutaredoxin GLRX5 dimer and synthesized by cytoplasmic iron-sulfur assembly [4Fe-4s] cluster [80, 81].

In addition to the Fe-S cluster, heme is an essential iron-containing compounds in the body, serving as a cofactor in the formation of key proteins such as hemoglobin, myoglobin, and cytochrome C, and is highly conserved, similar to the Fe-S cluster [82]. Heme is synthesized from Fe^2+^ and protoporphyrin IX through enzymatic reactions coordinated in the cytoplasm and mitochondria [83]. Not all iron entering mitochondria is used to synthesize Fe-S clusters or heme; excess iron is stored in mitochondria after binding with mitochondrial ferritin (MTFT) to maintain mitochondrial iron homeostasis. However, some studies have shown that MTFT overexpression affects the synthesis of Fe-S clusters and heme. Further exploration is needed to understand the regulation of mitochondrial iron content and the interaction among MTFT, Fe-S cluster, and heme [78, 84].

### Iron-induced cell death

Compared to that in the LIP, free iron preferentially enters mitochondria, making mitochondria primary storage sites for irons [85]. Iron deficiency can affect the synthesis of Fe-S clusters, heme, and other proteins, thereby interfering with normal cellular functions. Transition metal elements such as iron have strong redox activity, which is closely related to the production of ROS. ROS is the main factor of endogenous oxidative stress in cells and has potential harm to mitochondria [78]. The H_2_O_2_ produced by mitochondrial inner membrane protein complex I has strong hydrophilic and lipophilic properties, allowing it to permeate the unsaturated regions of the mitochondrial membrane. In a microenvironment with a high concentration of H_2_O_2_, free iron undergoes Fenton and Haber-Weiss reactions, which generate strong oxidizing products [86]. The following equations explain these reactions:

1 $${{\rm{Fe}}}^{2+}+{{\rm{H}}}_{2}{{\rm{O}}}_{2}\to {{\rm{Fe}}}^{3+}+{{\rm{OH}}}^{-}+{{\rm{HO}}}^{{{\bullet }}}$$

2 $${{\rm{O}}}_{2}^{-}+{{\rm{H}}}_{2}{{\rm{O}}}_{2}\to {{\rm{OH}}}^{-}+{{\rm{O}}}_{2}+{{\rm{HO}}}^{{{\bullet }}}$$

There is ongoing debate in the academic community regarding the conditions and specific process of the Haber-Weiss reaction, i.e., reaction (2) [87, 88]. However, free iron in mitochondria and the cytoplasm can generate highly oxidative HO•, which damages polyunsaturated fatty acids (PUFAs) in the phospholipid bilayer, leading to mitochondrial outer membrane permeabilization (MOMP), ultimately abrogating DNA stability and inducing cell death. Oxidative stress caused by iron can result in various forms of cell death, including apoptosis, pyroptosis, necroptosis, and ferroptosis [89, 90].

The intrinsic pathway of apoptosis is activated by many stressors [91]. When ROS-induced MOMP occurs, cytochrome C enters the cytoplasm and is linked to apoptotic protease-activating factor-1, followed by the activation and release of caspase-3/9, which in turn contributes to morphological changes such as apoptotic body formation, chromatin condensation, and DNA fragmentation [92]. Similar to apoptosis, pyroptosis and necroptosis are induced by caspases. In contrast to apoptosis, pyroptosis and necroptosis involve the leakage of cellular contents, which can activate the proinflammatory response and are classified as lytic forms of cell death.

Pyroptosis, mediated by gasdermin D (GSDMD), differs from other RCDs due to its caspase-1 dependence and inflammatory properties [93, 94]. When pathogen-associated molecular patterns (PAMPs) or damage-associated molecular patterns (DAMPs) stimulate the activation of the NF-κB signaling pathway, inflammasomes assemble. These inflammasomes bind to pro-caspase-1 that has been released into the cytoplasm, which is then cleaved into caspase-1, activating GSDMD and driving the release of inflammatory cytokines. Inflammatory cytokines cleave the N-terminal sequence of GSDMD, and this fragment binds to the cell membrane, forming pores and inducing cell rupture, ultimately leading to pyroptosis [95, 96]. The nonclassical pathways of pyroptosis mainly depend on caspase-4/5/11; these caspases are activated by direct interaction with inflammatory stimuli such as lipopolysaccharide (LPS), which cleave and activate GSDMD, initiating pyroptosis [95, 97].

Necrosis was previously defined as type III cell death and is widely recognized as a form of accidental cell death. It is a kind of RCD similar to necrosis in terms of cell morphology, which includes cell swelling, membrane rupture, chromatin condensation, and the induction of inflammatory mediators. The classical necroptosis pathway is also the tumor necrosis receptor pathway. Death receptors (e.g., TNFR and Fas), Toll-like receptors, and cytosolic nucleic acid sensors form an autocrine feedback loop [98], recruiting proteins such as TRADD and the linear ubiquitin chain assembly complex, which further interact with caspase-8 and RIPK1 to promote RIPK1 ubiquitination. After stimulation by the relevant signals, RIPK1 undergoes ubiquitination, recruits RIPK3, forms the RIPK1/RIPK3 complex, mediates MLKL oligomerization and forms specific necrosomes in the cytoplasm, thereby leading to pore formation on the plasma membrane [89] with cell swelling and membrane rupture. Activated RIPK3 can induce mtROS production by binding to the E3 subunit of the pyruvate dehydrogenase complex [99]. During infection, GSDMD binds to the mitochondrial membrane to form pores, releasing mtROS and promoting RIPK1/RIPK3/MLKL-dependent necroptosis [100].

In contrast to the aforementioned RCD types, ferroptosis exhibits higher dependence on transition metals, particularly iron, and it does not require caspase action but relies on the oxidative activity of Fe^2+^, which is its main distinguishing feature. The morphological changes in ferroptotic cells are largely concentrated in the mitochondria, including mitochondrial shrinkage, increased mitochondrial membrane density and rupture, and reduced mitochondrial cristae. Ferroptosis is essentially the outcome of oxidative stress caused by iron overload, with Fe^2+^ and PUFAs and lipid peroxidation the leading cause of ferroptotic cell death [101, 102]. PUFAs are added to phospholipids (PLs) via the esterification action of long-chain fatty acid CoA ligase 4 (ACSL4), which enters the membrane to generate PUFA-PLs. PUFAs react with the products of the Fenton reaction, producing phospholipid hydroperoxides (PLOOH) after dehydrogenation. Fe^2+^ is not only the leading participant in the Fenton reaction during ferroptosis but also causes ferroptosis through other programs. arachidonate lipoxygenases (ALOXs) catalyze the oxidation of PUFAs to generate hydroperoxy PUFA derivatives. Because ALOXs are a nonheme iron-containing enzymes, the presence of iron significantly increases their oxidative activity, and the subsequent generation of PLOOH continues to react with Fe^2+^, generating new lipid radicals. When the central repressors of ferroptosis, such as glutathione (GSH) and lipid enzyme glutathione peroxidase 4 (GPX4) [103], which participate in peroxide reduction and reduce product toxicity, show insufficient activity, the lipid radicals formed cannot be cleared and continue to generate new oxidative products via chain reaction. The accumulated peroxidized lipids eventually destroy the membrane structure and cause cell death [104].

Most of the iron-induced cell death is caused by ROS produced by Fenton reaction [90, 105, 106]. Interestingly, there are cases of iron-dependent death unrelated to ROS in fungi; notably, some fungi exhibit growth inhibition under high levels of cytoplasmic iron, and this phenotype is not associated with antioxidant enzymes [107]. Although these outcomes have been found only in fungi, they suggest unique relationships between metal ions and cell death and indicate that oxidation is not the only factor causing cell damage. Accumulating evidence has shown that some proteins involved in cell death, especially death receptors, are directly regulated by iron ions: the key death receptor Fas in apoptosis is expressed in two isoforms, an anti-apoptotic and a pro-apoptotic form, and iron is the key regulator in Fas exon splicing. An increase in iron content switches Fas from being an anti-apoptotic protein to being a pro-apoptotic protein, thereby activating the extrinsic apoptosis pathway and promoting necroptosis [91]. Nevertheless, the list of direct impacts of iron on cell death that have been discovered to date is still incomplete, and further exploration is required.

### Mitochondrial quality control and iron-induced cell death: a double-edged sword

Mitochondria are highly susceptible to the impacts of the iron-mediated Fenton reaction. Mechanisms underlying mitochondrial quality control include inhibition of ROS production within mitochondria, a decrease in the accumulation of abnormal proteins, and prevention of iron-induced cell death. For example, in the case of mitochondrial fusion, the overexpression of Fzo1A/B or MFN1/2 prevents excessive fragmentation of mitochondria. These fragmented mitochondria increase cellular sensitivity to apoptotic stimuli, while enhanced mitochondrial fusion significantly reduces the occurrence of MOMP, thereby inhibiting cell apoptosis [108]. Similarly, in cells under oxidative stress conditions, activated NRF2 can enhance the expression of MFN1 and MFN2 while degrading DRP2 through the proteasomal pathway, leading to mitochondrial hyperfusion, which temporarily protects cells and alleviates oxidative stress as well as inhibits ferroptosis [109]. Mitochondria undergoing imbalanced fusion and fission cannot maintain normal function for an extended period; when the accumulated damage exceeds the range tolerated for mitochondrial fusion, mitochondria are fragmented, triggering mitophagy to prevent further damage [110].

Even targeting the GSH/GPX4 antioxidant pathways may initiate several types of cell death, including ferroptosis. Many members of the HSP family can counteract oxidation through the FSP1/CoQ10 axis and other pathways; for example, HSP70 upregulates GPX4 expression to prevent ferroptosis induced by lipid oxidation [111]. HSP90 directly interacts with GPX4, inhibiting GPX4 activity and resulting in ferroptosis [112]. Additionally, HSP90 regulates signaling pathways such as the RIPK1 and RIPK3 signaling pathways; HSP90 inhibitors can significantly suppress necroptotic cell death [113].

**Fig. 4:**
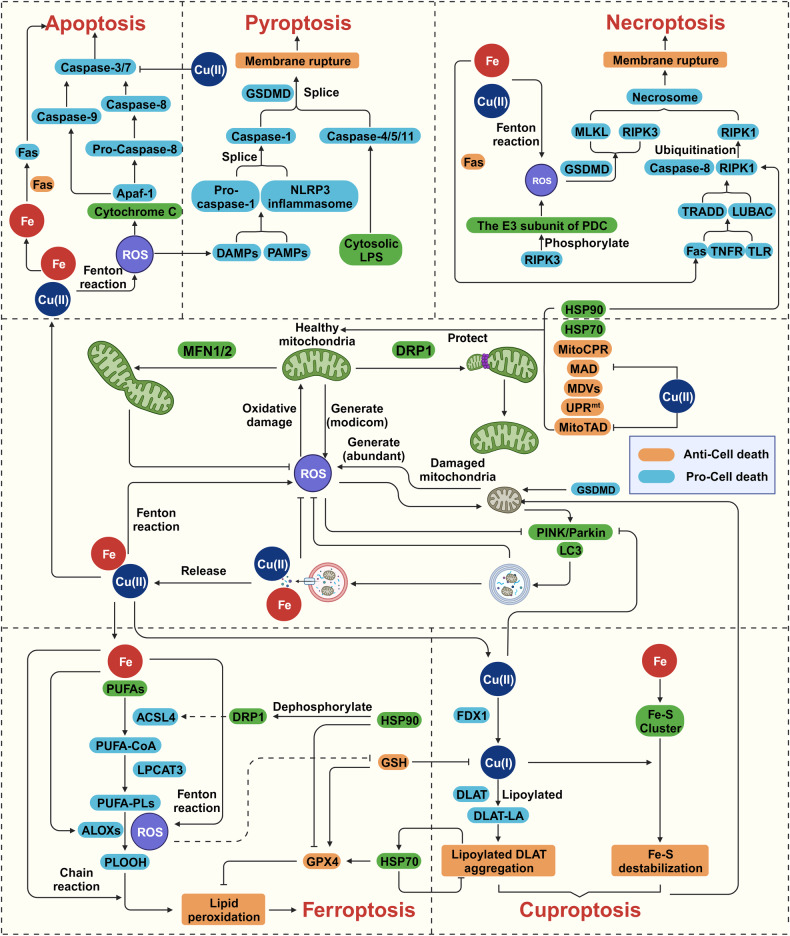
Mitochondrial quality control regulates cell death induced by copper and iron. Mitochondria are susceptible to ROS and they generate and maintain stability through quality control pathway. Excessive mitophagy will release metal ions in mitochondria, in which copper and iron undergo Fenton reaction and release a large amount of ROS, activating the intrinsic pathway of apoptosis, DAMPs-induced pyroptosis, and GSDMD-mediated necroptosis. Excessive iron ions and ROS react with PUFAs in a chain reaction, leading to uncontrollable lipid peroxidation and ferroptosis, or converting anti-apoptotic Fas into pro-apoptotic and promoting apoptosis or necroptosis. Copper ions induce cuproptosis by triggering protein lipoylation, or inhibit MAD and mitoTAD to aggravate mitochondrial stress and damage. The graph was created with BioRender.com. ACSL4 long-chain fatty acid CoA ligase 4, ALOXs arachidonate lipoxygenases, DAMPs damage-associated molecular patterns, DLAT dihydrolipoamide S-acetyltransferase, FDX1 ferredoxin 1, GSH glutathione, GPX4 glutathione peroxidase 4, GSDMD gasdermin D, LC3 light chain 3, LPCAT3 lysophosphatidylcholine acyltransferase 3, LUBAC linear ubiquitin chain assembly complex, NLRP3 NLR family pyrin domain containing 3, MAD mitochondrial-associated degradation, MitoCPR mitochondrial damaged protein import response, MitoTAD mitochondrial protein translocation-associated degradation, MLKL mixed lineage kinase domain like pseudokinase, PAMPs pathogen-associated molecular patterns, PLOOH phospholipid hydroperoxides, PUFA polyunsaturated fatty acids, RIPK3 receptor interacting serine/threonine kinase 3, ROS reactive oxygen species, TLR Toll-like receptors, TNFR TNF receptor, TRADD TNFRSF1A associated via death domain, UPR^mt^ mitochondrial unfolded protein response.

Mitochondria are among the primary sources of ROS [114]. Compared to those of the mitochondrial quality control system, the characteristics of mitophagy, which completely degrades mitochondrial quality control system substrates, make it the ultimate program to manage damaged mitochondria, playing inhibitory and remediating roles in iron-induced cell death, such as ferroptosis (Fig. 4). Experimental evidence showed that activating mitophagy through genetic or pharmacological approaches, especially in the early stages of oxidative stress, can significantly reduce the risk of ferroptosis by clearing and degrading damaged mitochondria, possibly by preventing subsequent metabolic abnormalities from generating excess ROS [115]. Some studies indicated that mitophagosomes formed by mitophagy not only isolate abnormal mitochondria but also serve as new iron storage spaces in mitochondria, preventing Fenton reaction-generated ROS from inducing further cell death [116]. However, whether Fenton reaction affects the mitophagosomes themselves is unclear.

Following the induction of oxidative stress, ROS levels are increased and these abundant ROS activate mitophagy, which is a remedial measure to prevent cell death [116]. Tang et al. found that in human pancreatic ductal adenocarcinoma (PDAC) cell lines, treatment with erastin induced STING1 translocation from the ER to mitochondria and triggered mitochondrial fusion by binding to MFN1/2, which led to increased mtROS levels without triggering PINK1/Parkin-independent mitophagy, eventually causing ferroptosis [117]. Moreover, mitophagy is not omnipotent; the number of ROS produced by damaged mitochondria may exceed’ the processing capacity of the mitophagy machinery, and in some cases, mitophagy may even promote cell death. First, the potential roles of mitophagosomes in isolating mitochondrial iron are likely temporary measures. Thus, iron will eventually be released in large quantities with the degradation of mitochondria, triggering the Fenton reaction and inducing an increase in mitophagy-dependent ROS levels [118, 119]. Interestingly, mitophagy plays a role in recycling iron from mitochondria in iron deficient cells, and iron chelators induce PINK1/Parkin-independent mitophagy in these cells [120]. A similar phenomenon has been observed in the SH-SY5Y neuroblastoma cell line, in which autophagy induced by deferoxamine inhibited apoptosis [121]. However, experiments have shown that mitochondrial membrane potential at this time point is not significantly damaged. The criteria for mitochondria to be iron sources in this process and how to stop the reaction when normal iron concentrations are reached require further exploration. Second, in addition to Fenton reaction mediation, iron exerts a strong effect on cell death programs, such as by cleaving Fas and other death receptors or stimulating oxidation of the mitochondrial outer membrane protein Tom20, which triggers pyroptosis [122]. Moreover, iron exerts an inhibitory impact on various antioxidant pathways; for example, apurinic/apyrimidinic endodeoxyribonuclease 1 (APEX1) engages in DNA repair and redox-regulating activities to activate transcription factors, thereby playing a crucial role in combating oxidative stress [123]. APEX1 inhibitors induce cell death, such as apoptosis and pyroptosis [124], while iron significantly inhibits APEX1 activity, exacerbating the damage to cells caused by oxidative stress [125]. To date, we have been unable to determine the relative impact of mitophagy on cell death pathways in terms of iron release and ROS level reduction, as the outcomes seem to vary markedly under different conditions, such as differences in tissue type, cell type, and physiological state. When antioxidant mechanisms such as GPX4 are defective, ATG3 and ATG7 undergo irreversible oxidation, inhibiting LC3 activity during autophagosome maturation and suppressing autophagy [126]. Taken together, mitophagy plays a dual role in iron-dependent cell death: under physiological states or the early stages of pathogenesis, including the development of oxidative stress, mitophagy degrades damaged mitochondria through negative feedback signaling, preventing ROS generation and hindering the initiation of iron-dependent cell death; however, under specific circumstances, persistently activated mitophagy can temporarily decrease mitochondrial-derived ROS levels while releasing excessive levels of iron, which accelerates the Fenton reaction and impairs the normal function of related proteins, forming a vicious cycle that ultimately leads to irreversible iron-dependent cell death.

Mitocytosis is another mechanism in which mitochondria are eliminated from cells, and it differs from mitophagy. Notably, free mitochondria and mtDNA can cause damage to surrounding tissues in some cases [127]. Although mitochondria excreted by migrasomes are enveloped by membrane structures, the influence of mitocytosis on cells and tissues and how the body clears these waste products remain unknown [74]. Fang et al. found endocytic transcriptome signatures and high TREM2 expression in the cardiac CD163^+^RETNLA^+^(Mac1) macrophage subpopulation. TREM2-overexpressing Mac1 cells engulfed exospheres containing cardiomyocyte-derived mitochondria outside cardiomyocytes and reduced cardiomyocyte injury in the context of sepsis [128]. These results do not indicate a direct link between these processes and mitocytosis, but there is no doubt that the removal of extracellular mitochondrion-containing vesicles may be involved.

## Mitochondria and other metals

### Mitochondria and copper metabolism

As an essential trace element, copper is obtained mainly through the digestion of food, followed by absorption in the intestines, and transport into cells by DMT1 and transmembrane copper transporter 1 (Fig. 3) [129]. Based on the electron configuration of transition metals, cytosolic copper forms various complexes, which constitute proteins such as albumin and ceruloplasmin for transportation among organs and tissues or serve as cofactors for key metabolic enzymes in physiological responses [130, 131]. Cytosolic copper enters mitochondria through the cytosolic anionic ligand (CuL) complex or binds to copper chaperone proteins, including copper chaperone for superoxide dismutase (CCS), human antioxidant protein 1, and cytochrome c oxidase copper chaperone 17, before being transferred to copper-storing proteins such as COX1, copper metallothionein 1, and superoxide dismutase [Cu-Zn] (SOD1). Most of these processes occur in mitochondria [132, 133]. Copper proteins and CuLs in the cytosol and mitochondria serve as copper stores and are essential regulators in maintaining physiological functions, such as OXPHOS, iron mobilization, and pigment formation [134, 135]. Excess copper in cells is primarily transported out of the cytosol by two copper-ATPases, ATP7A and ATP7B. When cytosolic copper levels exceed physiological levels, ATP7A and ATP7B, normally stored in the trans-Golgi network, translocate to the cytoplasm and fuse with the plasma membrane, from which it is released [136].

### Copper-induced cell death

Copper ions, especially Cu^+^, generate highly oxidative ROS through Fenton and Haber-Weiss reactions, similar to iron [137]. Cu^+^ catalyzes the formation of ·OH and ^1^O_2_ from H_2_O_2_, and is oxidized to yield Cu^2+^, which rapidly depletes intracellular GSH levels and decreases the antioxidant capacity of the cell. The reaction between Cu^2+^ and GSH regenerates Cu^+^, exacerbating intracellular oxidative stress and forming a vicious cycle [138]. Copper also contributes to changes in related proteins, such as the downregulation of Bcl2 and upregulation of caspase1/3/8 [139]. Thus, copper induces most cell death programs that are also triggered by iron, including apoptosis [140], pyroptosis [141], necroptosis [142], and even ferroptosis [143], although copper chelators effectively inhibit the death responses [144]. Interestingly, as an essential component of the antioxidant enzyme system, SOD1 exhibits the ROS-scavenging function that relies on copper delivered via the molecular chaperone CCS. SOD1 maintains intracellular copper levels within a specific range and ensures cell antioxidant capacity, which is maintained without causing excessive oxidative damage.

The damage induced by copper to cells is not apparently be limited to oxidative stress. Antioxidants such as α-tocopherol have not achieved the expected effects on copper-induced cell death, and the cytotoxicity of copper ion carriers may depend on copper itself [8]. Tsvetkov et al. investigated related targets of copper and related mitochondrial functions using copper ionophores such as elesclomol and discovered a copper-induced specific RCD, which they named cuproptosis [9, 145]. Based on recent experimental results, compared to ferroptosis, cuproptosis depends more profoundly mitochondria, especially the TCA cycle within mitochondria. Cu^2+^ Accumulated in cells enters mitochondria and is reduced to Cu^+^ under the action of FDX1, upregulating mitochondrial TCA-related proteins such as lipoylated DLAT while also directly binding to them, subsequently producing form long protein chains and aggregates. Aggregated lipoylated proteins in mitochondria induce substantial toxicity, and FDX1 affects the stability of Fe-S cluster proteins, leading to proteotoxic stress and even cell death [9, 146]. Although the mechanism regulating proteotoxic stress and the close connections between cuproptosis and mitochondrial oxidative respiration remain unclear, the aforementioned results revealed a new form of cell death independent of oxidative stress.

Copper and copper complexes can induce paraptosis-like death in human cancer cells through ROS-independent pathways [147]. Paraptosis is an RCD characterized by swelling of mitochondria and/or the ER accompanied by cytoplasmic vacuolization, relies the MAPK family proteins but not caspase activation [148]. Copper complexes, such as the Hinokitiol copper complex, inhibit the deubiquitinase activity of certain proteasomes, contributing to ATF4-associated ER stress and subsequently to paraptosis [147].

### Mitochondrial quality control and copper-induced cell death: a diverse relationship

The ability of mitochondria to turnover and store copper makes them highly sensitive to copper concentrations. Both copper deficiency and copper in excess affect the number and morphology of mitochondria to a certain extent, and these effects are related to the activity of respiratory complex IV and the expression of dynamic mitochondrial proteins. The function of respiratory complex IV is regulated via its copper-binding site. When copper concentrations are low, the expression of MFN1/2 and OPA1 is increased, thereby augmenting mitochondrial fusion to form giant mitochondria that can compensate for insufficient OXPHOS. As the copper concentration increases, the expression levels of fusion-related proteins begin to decrease, and DRP1 expression gradually increases. Noncytotoxic copper overload induces physiological levels of ROS production, which, to some extent, stimulates mitochondrial biogenesis. The restored function of respiratory complex IV plays a role in mediating mitochondrial fission [135]. Cells exposed to high concentrations of copper for an extended period have a larger volume and more mitochondria to ensure normal osmoregulatority [149]. Of course, when copper concentrations exceed the physiological range, mitophagy is involved, aggravating mitochondrial membrane damage and functional impairments [139]. Additionally, copper participates in the autophagy process, as it is essential for the activity of autophagy kinases, including MEK1/2 and ULK1/2 [150].

Based on the findings mentioned above, an organism initiates different mitochondrial quality control pathways at various copper concentrations to maintain physiological function and prevent cell death. However, certain contradictions challenge our understanding the potential underlying mechanisms (Fig. 4). First, although copper is essential for autophagy, high concentrations of copper might induce mitophagy by generating mtROS [151–153], altering mitochondrial membrane potential or inhibiting mTOR activity [154, 155]. Cells reduce the copper content through this negative feedback way of copper-induced mitophagy to maintain homeostasis, but studies have shown that copper has an inhibitory effect on autophagy. High copper concentrations result in the degradation of PINK1 and Parkin or inhibit LC3 expression, preventing the binding of substrates to autophagosomes and subsequently leading to apoptosis or pyroptosis [141, 149]. Moreover, copper induces lysosomal sedimentation, preventing the degradation of mitochondria enclosed in autophagosomes and hindering continuous mitophagy progression [156]. It is currently unclear how these processes are precisely modulated, as the degradation or inhibition of related proteins does not occur synchronously even when copper ion concentrations are similar. Parkin degradation may be mediated in conjunction with increases in LC3 expression, which may be affected by differences in cell types or copper ion carriers; in any case, the specific mechanism needs to be investigated further. Additionally, other molecules involved mitochondrial quality control can be influenced by copper. Under oxidizing conditions, copper ions inhibit cytosolic mitoTAD and MAD key enzymes Cdc48, while the expression of matrix protease CLpP is related to ATP7A levels [157, 158]. However, it is unclear whether these relationships are associated with cell death. Second, the mechanisms underlying copper-induced cell death are poorly understood. Copper seems to inhibit the activity of enzymes necessary for the execution of specific cell death processes, such as caspase-3 inhibition in paraptotic cell death induced by copper, hindering apoptosis [159]. The same phenomenon also occurs in cuproptosis [146]. Whether mitochondrial quality control can restore the function of aggregated lipoylated proteins or other copper-affected enzymes in the pathogenesis of cuproptosis, as some regulatory systems, including the UPS, may be inhibited by copper [147]. Third, the connections between copper-induced cell death and the TCA cycle make their respective networks with mitochondrial quality control even more complex. It has been reported that moderate prehypoxia exposure significantly increases TCA cycle enzyme activity and cellular antioxidant capacity and the mitophagy rate to resist stress caused by copper, while excessive ROS production accelerate glycolysis and decrease TCA cycle activity [160]. Notably, ROS production mediated by the Fenton reaction is inevitable when cells are exposed to a high copper level. In these cases, on the one hand, does the reduction in the TCA cycle change the rate of cuproptosis induction? On the other hand, what impact do changes in mitochondrial dynamics induced by increased copper level exert on cuproptosis? Does an increased mitochondrial biogenesis and/or fission rate lead to the production of more TCA-related proteins to properly transport or store more copper, or are more substrates for the cuproptosis reaction produced?

Cuproptosis provides new evidence that metal-induced cell death is not solely dependent on oxidative stress, and its close relationship with mitochondria gives us a further understanding of the role of mitochondrial quality control in cell death. Considering that cuproptosis-related proteins DLAT, LIAS, FDX1, and lipoylated mitochondrial enzymes are in mitochondria, the treatment of MAD, mitoCPR and mitoTAD outside mitochondria and on the surface of mitochondria may not play a role. It seems that mitophagy and other cell programs can directly prevent the production of toxic lipid metabolites, and some experts speculate that mitochondrial quality control might inhibit cuproptosis [8]. Theoretically, lipoylated mitochondrial components can be eliminated by lysosomal degradation pathways such as MDV, mitochondrial fission, mitophagy, and mitocytosis. However, considering the connections among mitophagy, ferroptosis, and cuproptosis pathways, we cannot rule out the possibility that mitophagy and other “last resort” responses are counterproductive in combating cuproptosis. The degradation of mitochondria, which serve as copper pools, may release increased levels of copper, similar to the release of iron during ferroptosis. Excessive degradation of mitochondria and excessive release of metals may cause oxidative stress-induced death such as ferroptosis through Fenton reaction. On the other hand, under high copper conditions, we cannot determine whether the remaining mitochondria can maintain a normal energy supply and avoid their fate as cuproptosis executors. UPR^mt^ is an important way to remove abnormal proteins in the mitochondrial matrix, which may be the key to inhibit copper death. It should be noted that UPR^mt^ handles misfolded proteins through molecular chaperones and matrix proteases in mitochondria, but whether lipoylated mitochondrial enzymes are within this range is unknown. Unfortunately, there are few studies related to cuproptosis, and most of them are based on bioinformatics analysis, which makes us still know little about cuproptosis. Here, we raise several questions to provide a reference for research on cuproptosis. First, do cells possess pathways that inhibit cuproptosis, and can they recognize and remove lipoylated mitochondrial enzymes? Second, due to the TCA cycle, is cuproptosis accompanied by changes in membrane potential, which is important for inducing mitochondrial fission and mitophagy? Third, is the role of mitophagy in cuproptosis similar to that in ferroptosis? It is urgent to further examine the mechanism of cuproptosis and its relationship with mitochondrial quality control, which may provide new ideas for the prevention and treatment of diseases caused by copper metabolism disorders.

### Other metals

Most metal ions, such as manganese, calcium, and zinc ions, play essential roles in synthesizing mitochondrial proteins. Abnormal levels of these ions appear to be associated with cellular dysfunction and even cell death [161–163]. Zinc and manganese are crucial cofactors in forming mammalian SOD (Fig. 3); the former cooperates with copper in synthesizing cytoplasmic SOD1, while the latter constitutes mitochondrial SOD2. Notably, the mitochondrial inner membrane protein Oma1, lipid transfer protein Yme1L, and energy metabolism-related proteins such as COX and Atp32 are involved in mitochondrial dynamics. The synthesis of these proteins requires the cytoplasmic zinc. Zinc deficiency impairs the antioxidant capacity of SOD1 and induces the p53 pathway to cause ROS accumulation, leading to apoptosis [164]. Supplementation of cells with zinc obviously reverses this process, while excess zinc triggers oxidative stress and ROS production, which further induces the oxidation of zinc-binding proteins and the release of zinc, forming a positive feedback loop that accelerates cell death [165]. In addition to GPX4, xCT, etc., DHODH on the surface of mitochondria exerts a protective effect on ferroptosis. Excess manganese downregulates DHODH together with the induction of ferroptosis [166].

Calcium is critical for signal transduction, and it enters mitochondria via osmosis or the action of channels and proteins such as Ca^2+^ uniporter complexes transport it [167]. Increased calcium and ROS levels activate the mitochondrial permeability transition pore, disrupt mitochondrial membrane potential and change the osmotic pressure, and ultimately result in necroptosis [168]. Moreover, excessively high calcium levels in mitochondria induce the activation of calmodulin-dependent protein kinase II delta (CaMKIIδ) and the RIPK3/MLKL cascade, while overexpression of MFN2 significantly inhibits necroptosis [169].

Clearly, imbalances in these metal levels might lead to various forms of cell death. Unfortunately, we have not found a highly specific metal-induced cell death similar to ferroptosis and cuproptosis. In recent years, new types of metal ion-induced cell death, however, have been identified, and some of these death modalities are specific to a regulatory pathway. Excess zinc triggers nonapoptotic cell death by causing mitochondrial swelling and inhibiting normal ATP synthesis [170, 171]. Calcium overload affects normal calcium signaling, influencing cell death, and the concept of “calcicoptosis” has been proposed [172]. Since mitochondria are critically involved in calcium ion transport and zinc participates in mitochondrial ETC and ATP synthesis, an imbalance in calcium and zinc ions can substantially damage mitochondria. Nonetheless, whether these two types of cell death exhibit specificity and can be considered novel forms of death on the basis of the Nomenclature Committee on Cell Death, similar to ferroptosis, warrants further investigation.

## Relevant clinical diseases

### Neurodegenerative diseases

Generally, neurodegenerative diseases (NDDs) exhibit multiple and common pathological effects, including defects in mitochondria, protein quality control, and the autophagic-lysosomal network, which lead to the aggregation and accumulation of misfolded proteins [173, 174]. Additionally, metal ion metabolism disturbances are the basis for the onset of various neurological diseases [175]. For instance, mutations in PRKN and PINK1 are often evident in patients with familial and sporadic Parkinson’s disease (PD) (Table 1). The absence of PINK1 inhibits PINK1-dependent mitophagy, and although cells may clear mitochondria through other pathways, the efficiency of their elimination is reduced, resulting in the accumulation of damaged mitochondria, iron, and synuclein alpha (SNCA) or even ferroptosis in the substantia nigra of PD patients [176, 177]. Long-term exposure to metals, including copper, iron, and manganese, increases the risk of PD, possibly due to the elevation in ROS levels mediated by the Fenton reaction or the aggregation of SNCA [178, 179]. Interestingly, decreased copper content in the substantia nigra of PD patients has been noted, and the underlying mechanisms of this outcome require further elucidation [180]. Similarly, in Alzheimer’s disease (AD) models, activation of pathways such as the UPS and autophagy pathways is inhibited. In the early stages of AD, the number of autophagosomes increases, but these autophagosomes do not fuse normally with lysosomes, and toxic proteins, including amyloid β (Aβ), tau protein, and damaged mitochondria, cannot be cleared before they cause damage [181–183]. Due to the physiological demand, copper and zinc are abundant in cortical tissue, and in the development of AD, these metals can cause endogenous regulatory disorders, such as decreased ATP7B activity, thereby inducing metal-related abnormalities in the nervous system even without environmental metal exposure [184, 185]. Excessive metals levels increase the burden on mitochondria and promote the formation of Aβ as well as lipid peroxidation, damaging neurons and the blood-brain barrier. Friedreich’s ataxia (FRDA) is another common NDD. Frataxin, a protein involved in Fe-S cluster biosynthesis, reportedly plays a vital role in the pathogenesis of FRDA. The Fxn gene encodes Frataxin, and complete knockout of Fxn in mice caused cell apoptosis in early embryonic stages [186]. When frataxin was absent, Fe-S synthesis was blocked, which not only interfered with the normal ETC but also forced cells to compensate for these effects by taking up additional iron, leading to iron overload and oxidative stress and triggering mitophagy [187]. Upregulated expression of PGC-1α, a marker of mitochondrial biogenesis, has been observed in fibroblasts from late-stage FRDA patients, suggesting accelerated mitochondrial biogenesis [188].

**Table 1:** Relationship between mitochondrial quality control and various diseases associated with metal-dependent cell death.

Classifications	Diseases	Metal-dependent cell death	Main findings	References
Neurodegenerative diseases	Parkinson’s disease	Apoptosis, ferroptosis, pyroptosis	The loss of Parkin and PINK1 resulted in the inhibition of mitophagy, the enhancement of oxidative stress, and the accumulation of iron and SNCA in the substantia nigra. Exposure to copper and other metal ions inhibited the expression of Parkin and PINK1 and produced ROS via the Fenton reaction, promoting the release of NLRP3 and inflammasome.	[141, 176]
	Alzheimer’s disease	Apoptosis, necroptosis, pyroptosis, ferroptosis	The inhibition of the UPS pathway affected the control of mitochondrial protein content and the degradation of Aβ and tau protein. UPR^mt^ and mitophagy were inhibited, aggravating oxidative stress. The cortex prevented high metal ion levels from increasing mitochondrial burden and augmented Aβ formation and lipid peroxidation. Aβ aggravated mitochondrial stress and induced mitophagy-dependent ferroptosis.	[182, 183, 294, 295]
	Friedreich’s ataxia	Ferroptosis, apoptosis,	The absence of Frataxin blocked Fe-S synthesis, interfered with the electron transport chain, compensatory increases iron uptake and mitochondrial biogenesis, thereby leading to iron overload and oxidative stress together with mitophagy.	[187, 188, 296]
	Wilson disease	Apoptosis	ATP7B mutation caused copper metabolism disorder and activated autophagy by inhibiting mTOR. Inhibition of autophagy protein accelerated copper-induced apoptosis.	[297]
	Amyotrophic lateral sclerosis	Apoptosis, ferroptosis,	MiR-335-5p mutation or abnormal iron metabolism affected mitochondrial dynamics, contributing to mitophagy and apoptosis. The misfolded SOD1 was involved in forming large mitochondrial vacuoles and gradually developed into necroptosis.	[298–300]
Tumors	Melanoma	Necroptosis, ferroptosis, apoptosis	Inhibition of mitochondrial complex I promoted mitophagy and increased mitophagy-related ROS, eventually leading to necroptosis and ferroptosis. Inhibition of BNIP3-mediated mitophagy resulted in the upregulated ferritinophagy, increased iron levels in tumor cells, and induction of apoptosis in tumor cells.	[119, 301]
	Glioma	Ferroptosis, apoptosis	Alterations in HSP90 and DRP1 mediated the regulation of Acsl4 expression and promoted ferroptosis. Ultrasound stimulation and sonosensitizer were used to induce mitophagy, increase ROS and cause apoptosis of tumor cells.	[199, 302]
	Colorectal cancer	Cuproptosis, apoptosis,	Interfering with the Warburg effect enhanced the transformation of cancer cells from aerobic glycolysis to oxidative phosphorylation, resulting in cuproptosis in a high copper environment. Overexpression of PINK1 altered tumor metabolism, increased mitochondrial respiration, and caused cancer cell apoptosis.	[197, 303]
	Non-small-cell lung carcinoma	Ferroptosis	Erastin combined with celastrol destroyed mitochondrial membrane potential, augmented mitophagy and ROS production, and mediated ferroptosis in NSCLC. COX7A1 enhanced TCA cycle, OXPHOS, inhibited PINK1-mediated mitophagy and promoted ferroptosis in NSCLC in a high-level iron environment.	[200, 304]
Infection	Sepsis	Apoptosis, necroptosis, ferroptosis, pyroptosis	The failure of mitochondrial quality control made cells unable to resist inflammatory response and oxidative stress at the early stage of sepsis. Nutritional immunity resulted in an increase in intracellular metal ion levels, and cells were prone to multiple RCDs. Long-term uncontrolled mitophagy aggravated cell damage, and use of mitophagy inhibitors could reduce apoptosis during sepsis.	[208, 209, 305–308]
Vascular diseases	Thrombosis	Apoptosis, ferroptosis, pyroptosis	In patients with KD complicated by thrombosis, platelet mitophagy was increased, in turn promoting platelet activation and apoptosis. Platelet activation and abnormal iron metabolism accelerated thrombosis and induced oxidative stress as well as death in the vascular wall.	[220, 309, 310]
	Atherosclerosis	Apoptosis, ferroptosis, pyroptosis	Foam cells had abnormal lipid metabolism, and inhibition of autophagy aggravated atherosclerosis. Altering mitochondrial dynamics stimulated mitochondrial autophagy and abnormal cell proliferation of VSMCs, thereby aggravating atherosclerosis.	[224, 227]
	Ischemia-reperfusion	Apoptosis, necroptosis, ferroptosis, pyroptosis	Activation of the HIF signaling pathway to induce mitochondrial autophagy alleviated ferroptosis in AKI and abated kidney damage. Inhibition of FUNDC1-mediated mitophagy antagonized oxidative stress, apoptosis, and ferroptosis.	[236, 311–313]
Metabolic diseases	Diabetes	Apoptosis, ferroptosis	The β cells of diabetic patients are continuously exposed to high glucose environment, accompanied by iron overload. Due to the lack of antioxidant enzymes, mitophagy-related proteins are also inhibited, and sustained oxidative stress leads to β-cell apoptosis and ferroptosis, which further aggravates diabetes.	[244–250]
	NAFLD	Ferroptosis	Increased iron absorption and mitophagy inhibition in NAFLD patients lead to ferroptosis in liver cells in the early stage of the disease.	[258, 259]
Musculoskeletal diseases	Osteoporosis	Apoptosis, ferroptosis	ROS induces the increase of DRP1 in osteoblasts and osteoclasts and inhibits mitochondrial fusion, or further promotes ROS production by inducing mitophagy to release iron, causing apoptosis and ferroptosis.	[264–267]
	Osteoarthritis	Apoptosis, ferroptosis	Mitochondrial division of chondrocytes in OA patients increased, intracellular iron content increased, protein expression of GPX4 and SLC7A11 decreased, antioxidant capacity weakened, and apoptosis and ferroptosis were prone to occur.	[268–270]
	Sarcopenia	Apoptosis, ferroptosis	Aging SCs mitochondrial quality control and antioxidant capacity are weakened, and show age-related iron accumulation, causing apoptosis and ferroptosis.	[271–274]

Notably, aging is one of the main risk factors for most neurodegenerative diseases. Given the characteristics of permanent cells, neurons are more likely to accumulate DNA damage and exhibit changes in the epigenome and protein homeostasis, making them more sensitive to aging. In addition, aging cells show accumulation of metals such as copper and iron, often accompanied by impaired UPR^mt^ and autophagy [155]. Impaired mitophagy and/or ferritinophagy may be important factors in abnormal metal accumulation, but considering the differences in protein levels in different types of cells during aging, the interrelationship might be very complex, or it these relationships may be mutually reinforcing and causal [189]. In any case, aging cells exhibit high resistance to ferroptosis, which is due to long-term adaptation to iron accumulation or other unknown reasons [10].

Given the critical role played by mitochondria and metal ions in neurodegenerative diseases, maintaining the physiological range of mitochondrial quality control effects and metal ion concentrations in balance may be the key to preventing and treating neurodegenerative diseases. Experimental data have shown that metal chelators promote the dissolution of amyloid plaques in AD patients [190], and supplemental zinc modulates copper ion concentrations to some extent, affecting copper absorption and transport [191]. Restoring the normal response of mitophagy plays a protective role against the pathogenesis of neurodegenerative diseases. The antidiabetic drug repaglinide enhanced PINK1 expression, significantly inhibited cell apoptosis, and alleviated neuroinflammation and PD symptoms by activating mitophagy in a PD model [192]. These examples suggest novel strategies for the treatment of neurodegenerative diseases.

### Tumors

The physiological characteristics of tumor cells make them significantly different from normal tissue cells in terms of metal and energy metabolism. The growth of tumors is often accompanied by increased levels of metal ions such as copper and iron, and these metals participate in tumor cell proliferation, angiogenesis, and metastasis. When levels of metal ions are too low or chelating agents are used, the growth of tumors and angiogenesis are markedly reduced [193–195]. Most tumor cells undergo aerobic glycolysis not OXPHOS for energy supply, even under sufficient oxygen conditions, a mechanism known as the Warburg effect, to meet their unique energy and material metabolism needs. Tumor cells show significant increases in glucose uptake and lactate production. The individual tumor microenvironment produced via the Warburg effect clearly reduces the incidence of various types of cell death. Lactic acid makes the tumor environment weakly acidic, which is not conducive to the iron-mediated Fenton reaction. Even when the intracellular iron content is increased significantly, ROS is not produced in large quantities, which prevents oxidative damage induced by the iron-mediated Fenton reaction [193]. Moreover, the switch to aerobic glycolysis from OXPHOS protects tumor cell mitochondria from damage caused by electron leakage, thereby reducing the potential sources of ROS. Moreover, the mitochondrial DNA content in tumor cells is lower than that in normal cells, which, together with the weakly acidic environment, prevents oxidative stress-induced cell death. Diminished glycolytic function also reduces the utilization of TCA cycle-related enzymes and FDX1-related genes in mitochondria, allowing tumor cells in high-copper environments to resist cuproptosis [196, 197]. In addition, local hypoxic, acidic, and low-glucose environments further augment energy metabolism reprogramming in tumor cells, which not only upregulates antiapoptotic proteins, including protein kinase B (Akt) and hexokinase 2 (HK2) but also inhibits the proliferation of T lymphocytes, facilitating tumor cell immune escape [198].

HSP90 and DRP1 are involved in regulating cell ferroptosis. Overexpression of HSP90 enhances DRP1 dephosphorylation and alteration in mitochondrial morphology and increases ACSL4-mediated lipid peroxidation, ferroptosis of glioma cells, and anticancer activity of erastin (Table 1) [199]. Drugs such as itaconate derivative 4-octyl itaconate and tumor suppressor p53 disrupt the balance of the tumor microenvironment by interfering with the Warburg effect, promoting cancer cells to shift from aerobic glycolysis to OXPHOS, and promoting the establishment of high copper environments that are conducive to cancer cell growth and cause damage to cancer cells through cuproptosis or other forms of oxidative stress-induced death [196, 197]. Some treatment methods are effective because they induce mitochondrial quality control while interfering with the tumor microenvironment-promoting factors. For example, the COX7A1 subunit of cytochrome c oxidase plays a vital role in modulating the physiological function of the ETC. Overexpression of COX7A1 enhances TCA cycle-related enzyme activity and accelerates OXPHOS in human non-small cell lung carcinoma (NSCLC) cells. COX7A1 also inhibits PINK1/Parkin and PGC-1α expression, hinders autophagic flux and mitophagy activation, and mediates ferroptosis in NSCLC, which can be promoted by high levels of Fe^2+^ [200]. Similarly, specific inhibitors of the cancer protein myoferlin and WJ460 can trigger cell cycle arrest in the G2/M phase and upregulate mitophagy-related genes in PDAC cells, thereby leading to cell death due to excessive Fe^2+^ and ROS levels [118].

### Infection

In the case of severe infection or uncontrolled inflammation, the metabolism of metal ions is significantly altered. Microbial growth and proliferation require an abundant iron supply, and changes in peripheral blood metal levels in the host constitute a defense mechanism against pathogens [201]. Copper is an essential trace nutrient for pathogens; however, high levels of copper can kill these microorganisms. After a host is infected with a pathogen, the peripheral blood copper level is significantly increased to meet the need to synthesize cytokines such as interleukin 2 to enable the body to achieve an efficient immune response [202]. The activation of cytokines promotes inflammation while inducing an increase in the level of hepcidin, which inhibits ferroportin activity and upregulates the synthesis of iron-storing proteins, reducing the release of intracellular iron [203]. Therefore, although high concentrations of cytokines inhibit the production of red blood cells and reduce the consumption of plasma iron, the generation of nontransferrin-bound iron required for pathogen growth also decreases, leading to an increase in copper content but a decrease in the iron level in peripheral blood [204]. Changes in the environment limit the proliferation of pathogens to a certain extent and affect host cells. IL-1α, HSPs, and other DAMPs or PAMPs, such as LPS, stimulate oxidative stress in cells, triggering DRP1 upregulation and translocation, followed by the activation of mitophagy, which exerts a protective response in the early stages of diseases [205–207]. When infection and inflammation persist, however, mitophagy and a selective autophagy with ferritin serving as a substrate are simultaneously activated, and they increase free iron levels released from cells and mediate an uncontrolled cascade reaction, leading to the death of many immune cells and sepsis-induced immunosuppression (Table 1) [208, 209]. Additionally, activated macrophages release a large amount of ferritin during sepsis through nonclassical secretory pathways. These ferritins act as proinflammatory cytokines and form a positive feedback loop that increase inflammation and immunosuppression, causing hyperferritinemic sepsis [210–212].

### Vascular diseases and related diseases

The bidirectional effect caused by mitochondrial quality control is not unique to infectious diseases such as sepsis. Vascular diseases involve multiple tissues and organs, and thrombosis is the basis of various vascular diseases. Mitochondrial quality control and iron metabolism are important in thrombosis. High levels of ROS can directly stimulate platelet activation and aggregation or can activate platelets through the cell adhesion factor P-selectin [213]. Interestingly, each of these processes can be inhibited by iron chelators [214]. Additionally, GPXs downregulate platelet-dependent thrombosis, and GSH consumption induced by lipid peroxidation attenuates this response [215]. Although mitophagy limits iron release to a certain extent, the impact of the iron that is released remains unknown. A study showed that improving platelet energy and material supply function by mitophagy promoted platelet activation (Table 1) [216]. Kawasaki disease (KD) is a common self-limiting form of pediatric vasculitis that often involves coronary arteries due to thrombosis. Vascular smooth muscle cells (VSMCs) in children with Kawasaki disease can increase ROS levels due to abnormal autophagy processes, activating corresponding cell death pathways and upregulating expression of NLRP3, to exacerbate mitochondrial dysfunction and vascular inflammation [217]. Studies revealed that serum iron levels are often reduced and serum ferritin levels are abnormally high in patients with KD, and these effects can be mediated by hepcidin treatment [218, 219]. Through the combined action of various factors, VSMCs undergo iron overload and death, affecting vascular endothelial integrity. In addition, thymic stromal lymphopoietin is significantly elevated in patients, and it induces platelet activation via mitophagy agonists, contributing to thrombosis [220].

Atherosclerosis is another common disease caused by abnormal VSMCs and platelets; it is closely related to mitochondrial quality control in various cells and is often accompanied by apoptosis, pyroptosis, and ferroptosis of macrophages as well as endothelial cells [221]. Genetic or environmental factors lead to the accumulation of cholesterol-enriched lipoproteins in the arterial wall. Mitochondrial dysfunction caused by defects in long-term quality control mechanisms can dysregulate the normal function and damage the structure of VSMCs, stimulate oxidative modification of lipoproteins in the vascular wall, activate the immune response to recruit monocytes, and induce cells to differentiate into macrophages that engulf retained lipoproteins [222, 223]. Macrophages or smooth muscle cells that accumulate too much lipid are transformed into foam cells, which are more susceptible to iron overload [224]. Inhibition of autophagy in cells under high-fat conditions aggravates atherosclerosis, and iron accumulation often occurs in plaques, which may be caused by dysregulated mitophagy and the abrogation of the fragile metabolic balance in foam cells [225, 226]. Notably, mitochondrial quality control does not play a protective role in atherosclerosis. Experimental results demonstrated that apelin-13, an endogenous ligand of the G protein-coupled receptor angiotensin II protein J (APJ), increased DRP1 expression, inhibited fusion-related protein expression, and stimulated VSMC mitophagy and abnormal proliferation, which exacerbated the development of atherosclerosis [227]. Cells on the vascular wall continued to proliferate, aggregate, die, and accumulate to form atheromatous plaques. When the plaque ruptured, platelets form thrombi block the lumen, which is naturally narrow, subsequently causing ischemia and hypoxia in affected organs.

Hypoxia promotes the conversion of cells from aerobic respiration to anaerobic respiration. In addition to stimulating calcium overload caused by ATP enzyme imbalances, such as imbalances in calcium pump activity, metabolites, including lactic acid and succinic acid, accumulate, antioxidant enzyme activity is inhibited, the mitochondrial structure is destroyed and mitochondrial function is dysregulated under ischemic conditions. After blood flow recovery, mitochondrial permeability increases because of the high-level calcium, and moreover, succinate, mtDNA, and other substances are released. As DAMPs, these secreted factors activate the apoptotic pathway and autophagy and induce oxidative stress, and the antioxidant mechanism cannot be restored before apoptosis and ferroptosis are induces [228]. The heart is vulnerable to damage caused by metal overload and mitochondrial dysfunction due to the metabolic characteristics of cardiomyocytes [225, 229]. Most studies suggest that mitophagy exhibits a protective effect on the myocardium. Overexpression of Drp1 and Atg5 in cardiomyocytes or inhibition of p53 to increase autophagic flux can prevent symptoms such as myocardial hypertrophy and aging [230, 231]. In contrast, hypoxia stimulates FUNDC1-mediated mitophagy, but this process does not play a protective role after paraquat exposure, and it aggravates ferroptosis and apoptosis in cardiomyocytes [232]. After treatment with the mitochondrial inhibitor mdivi-1 or knocking down BNIP3, the myocardial infarct size related to mitophagy was significantly diminished [233, 234]. A similar response was found in the case of renal injury following an ischemia-reperfusion episode. Ischemia-reperfusion injury-activated mitophagy establishes a positive feedback loop involving apoptosis with proteins such as MEG3, which aggravates acute kidney injury that is secondary to ischemia-reperfusion injury [235]. Obviously, similar to the results of infectious diseases, mitochondrial quality control, e.g., activation of mitophagy, can indeed confer protection to blood vessels, myocardium, and kidney cells, but the damage caused by mitochondrial quality control is unpredictable [236]. Ways to make rational use of this response to control disease activity within a limited range, a remarkable challenge, need to be identified.

### Metabolic diseases

Changes in modern lifestyles and eating habits have led to a sharp increase in patients with metabolic diseases [237]. Obesity and diabetes are the most common types of metabolic diseases. These diseases can lead to a variety of related diseases, including cardiovascular disease and cancer, which impose great burdens to health system worldwide. Obesity is one of the main risk factors for metabolic diseases such as diabetes and non-alcoholic fatty liver disease (NAFLD) and is often accompanied by abnormal mitochondrial function and mitochondrial quality control. Compared with that in healthy individual, the expression of citrate synthase, which is the rate-limiting enzyme in the TCA cycle, is reduced in obese patients, which inhibits glucose and lipid metabolism and the normal energy supply of mitochondria and increases ROS production [238]. The expression levels of the mitochondrial fusion proteins MFN2 and OPA1 and the mitophagy-related protein p62 in obese patients is also significantly decreased, resulting in an imbalance in mitochondrial fusion, fission and clearance, which seriously affects mitochondrial quality [239–241]. In addition, a high-fat diet can significantly increase the level of caspase-3 and decrease the level of bcl-2, which increases the susceptibility of cells to apoptosis and causes a series of obesity-related complications [242, 243].

In addition to obesity, diabetes is another common metabolic disorder, and damage to islet β cells is the main cause of diabetes. The physiological characteristics of β cells and the high glucose environment associated with diabetes make these cells highly susceptible to death induced by iron and other metal ions. Previous studies have shown that in diabetic patients, the pancreas, especially islet β cells, is often characterized by excessive iron accumulation, and the level of antioxidant enzymes in β cells is low, which makes these cells susceptible to oxidative stress [244, 245]. Iron overload decreases glucose oxidation and increases fatty acid oxidation, which gradually leads to insulin resistance. Increased blood glucose levels induce excessive mitochondrial fission or fragmentation, thereby affecting OXPHOS, inhibit the expression of PINK1 and Parkin and downregulate the expression of genes such as PGC-1α, thereby inhibiting mitochondrial biogenesis and mitophagy and preventing cells from eliminating abnormal mitochondria. This increases ROS production and lipid accumulation through peroxidation, resulting in apoptosis and ferroptosis [246–248]. Recent studies have shown that the CLEC16A gene plays a protective role in type 1 diabetes by regulating β-cell mitophagy. The Clec16a protein encodes an E3 ubiquitin ligase, and the complex of this factor with Nrdp1 and Usp8 can promote the fusion of mitophagosomes and lysosomes. In an inflammatory state, knockout of Clec16a in β cells can significantly increase apoptosis and sharply increase blood glucose levels in patients, and hyperglycemia further affects mitochondrial quality control, promotes cell damage, and exacerbates diabetes. The accumulation of human amylin in pancreatic islets is a typical feature of type 2 diabetes. Amyloid overexpression stimulates mTORC1 signaling, inhibits mitophagy, and increases apoptosis. Amyloid protein aggregates can also form cytotoxic oligomers, destroy the integrity of the cell membrane, and further aggravate islet damage [249, 250]. A high-glucose environment affects islet β cells and mitochondrial quality control in the nervous system, cardiovascular system, urinary system cells and gradually causes common complications such as diabetic peripheral neuropathy, diabetic cardiomyopathy and diabetic nephropathy.

NAFLD is a common complication of obesity and diabetes. Excessive triglyceride and glucose levels can place a burden on the liver and cause liver fat infiltration. The accumulation of fat in the liver gradually progresses from initial steatosis to steatohepatitis and eventually progresses to cirrhosis [251]. Mitochondrial dysfunction and metal metabolism disorders are important causes of NAFLD [252]. Hyperferritinemia and liver iron deposition occur in NAFLD patients, which may be related to the increase in intestinal iron uptake and the decrease in liver cell iron efflux in NAFLD patients [253, 254]. During the early stage of NAFLD, liver cells exhibit increases iron levels, oxidative stress and ferroptosis-related phospholipids and decreased mitopahgy [255–257]. The use of ferroptosis inhibitors can reduce hepatocyte death and inflammation during NAFLD and alleviate the progression of NAFLD. In addition, the key ferroptosis factor frataxin not only plays a role in neurological diseases but is also involved in the occurrence and development of NAFLD. Early studies confirmed that frataxin deficiency could lead to obesity in mice. In the response to a high-fat diet and free fatty acids, the level of frataxin in the liver is significantly reduced. Activation of frataxin can enhance PINK1/Parkin-mediated mitophagy, which can significantly ameliorate lipid accumulation induced by a high-fat diet and free fatty acids [258, 259].

Iron is not the only metal ion involved in metabolic diseases. Abnormal Ca^2+^ levels caused by mitochondrial Ca^2+^ uptake disorders have also been shown to be involved in the pathogenesis of type 2 diabetes [260]. In addition, higher serum copper levels are associated with obesity and diabetes, and increased copper levels have been observed in the liver cells of NAFLD patients, which suggests that copper can induce these metabolic diseases. However, in some interventional studies, copper has been shown to exert a protective effect on diabetic patients, and a restricted copper diet can also induce steatosis in the liver [261–263]. The specific role of copper and other metals, their ability to induce cell death in metabolic diseases and the mechanism need to be further studied.

### Musculoskeletal diseases

Bones and muscles maintain bodily functions such as breathing, eating and movement. Damage to these tissues can seriously affect patient quality of life and may cause more serious diseases and endanger life. Osteoporosis (OP) is a common motor system disease caused by an imbalance between bone resorption and bone formation. Mitochondria are the key factors that maintain the balance of activity between osteoblasts and osteoclasts. Abnormal mitochondrial quality control can lead to changes in the activity of osteoblasts and osteoclasts. An increase is ROS caused by various factors stimulates the expression of DRP1 in osteoblasts. Abnormal mitochondrial fission leads to mitochondrial dysfunction or fragmentation, which affects the function of osteoblasts. Moreover, abnormal mitochondria further aggravate the production of ROS, resulting in positive feedback. However, in osteoclasts, DRP1 overexpression promotes osteoclast differentiation [264]. In addition, when the proportions of S-OPA1 and L-OPA1, which mediate mitochondrial fusion are out of balance, mitochondrial fusion in osteoblasts is inhibited, and abnormal mitochondria produce large amounts of mtROS, thereby inducing osteoblast apoptosis [265]. Abnormal iron metabolism in mitochondria can also aggravate the progression of OP. A lack of MTFT induces mitophagy in osteoblasts through the ROS/PINK1/Parkin pathway to release iron, but excess the iron cannot be stored through the MTFT pathway, gradually accumulates and ultimately induces ferroptosis in osteoblasts [266]. However, some studies have shown that the absence of PINK1 leads to a significant reduction in bone mass in patients. How to regulate mitophagy to an appropriate level to maintain the balance between osteoclasts and osteoblasts is still a problem [267].

Osteoarthritis (OA) is a chronic disabling disease caused by articular cartilage and bone injury. Experimental results revealed obvious oxidative stress in chondrocytes during the development of OA, which was related to abnormal iron metabolism, mitochondrial metabolic disorders and other factors [268]. The proinflammatory cytokine IL-1β can increase TfR1 and inhibit FPN expression, promote iron transport into the cell and reduce iron efflux, and excessive iron causes mitochondrial dysfunction through the Fenton reaction. Furthermore, the expression of DRP1 was upregulated, resulting in the fragmentation of mitochondria and exacerbated oxidative stress [269]. In chondrocytes from mice with IL-1β-induced osteoarthritis, decreased protein expression of GPX4 and SLC7A11 and lipid ROS accumulation were observed, which inhibited the expression of type II collagen in chondrocytes and affected bone and joint health. Ferrostatin-1, which is a ferroptosis inhibitor, can alleviate these symptoms [270]. In chondrocytes from humans with osteoarthritis, IL-1β not only inhibited chondrocyte proliferation but also induced apoptosis. After treatment with α-ketoglutarate (α-KG), mitophagy flux increased, mitochondrial structure and function significantly improved, ROS production was inhibited, and OA was improved through the alleviation of chondrocyte apoptosis [268].

Satellite cells (SCs), which are located under the basal layer of muscle fiber, are critical for skeletal muscle regeneration, maintaining a reversible static state and activating myoblasts when needed. With age, SCs undergo irreversible senescence, which seriously affects the repair function of SCs and leads to sarcopenia [271]. A decrease in the number and function of SCs is associated with mitochondrial damage and metal-induced cell death. In aging SCs, mitochondrial fission, mitophagy efficiency, and the OXPHOS energy supply are low [272]. In a sarcopenia mouse model, muscle iron levels and ferroptosis markers were significantly increased, and GPX4 was downregulated. The use of the ferroptosis inhibitors Ferr-1 and DFO could significantly ameliorate these symptoms. In addition, age-related iron accumulation was observed in a model constructed with C2C12 myoblasts [273]. Experiments have shown that adiponectin, which is secreted by adipocytes, can significantly reduce the expression of PINK1 and Parkin induced by oxidative stress, thereby inhibiting mitophagy and apoptosis caused by mitophagy in C2C12 myoblasts, which provides a new idea for the diagnosis and treatment of skeletal muscle diseases such as sarcopenia and early-onset myopathies [274].

## Prospects and challenges

In recent years, with the discovery of cell death modes such as ferroptosis and copper death, we have gained a deeper understanding of metal ion-induced cell death. However, investigations are still in their infancy, and many scientific issues need to be resolved. Different metal ions exert shared influence on their transport and utilization. Various metal nutrients are obtained via intake of food, and most of them are transported from the intestine to intestinal epithelial cells or from the cells to the intestinal lumen, and both processes require DMT1. Inhibition of DMT1 expression reduces the transport of iron, copper, and zinc into cells, suggesting that DMT1 mediates the transport of multiple metals [275]. However, the number of transporters is limited and they can be saturated, and there is competitive inhibition between the transport processes of multiple metal ions [276]. When too much metal is ingested, the transport of other metals is affected. For example, silver plays a competitive role in mitochondrial copper absorption, significantly inhibiting the uptake of copper by mitochondria and influencing the subsequent COX assembly process [277]. In contrast, ceruloplasmin is involved in iron transport. Iron transport can be disrupted when copper levels are profoundly low or ceruloplasmin levels are too low, leading to gradual iron accumulation in tissues [160]. The specific impacts of the interaction between different metals on cells remain unclear, and various metal ions may be involved in the same cell death process. It has been reported that iron plays a role in copper-mediated ferroptosis, and the use of copper ion chelating agents does not abate iron accumulation in ferroptosis [278]. These findings raise new questions: what is the relationship between different cell death pathways in the presence of multiple metals, and how is the signaling that leads to the final death of the cell mediated? Unfortunately, in metal ion-mediated cell death, we have focus only on concentration changes of a certain metal ion and gave ignored the possible effects of other metal ions.

In addition, cell death induced by the overload of the same metal seems to be nonspecific; notably, death caused by metal-mediated oxidative stress is a common cause of a variety of types of cell death. Previous research results may provide possible explanations. First, identical proteins may exert opposite effects on different cell death pathways. For example, FSP1 is an NAD(P)H-dependent oxidoreductase that has long been regarded as a proapoptotic factor that synergistically triggers apoptosis with 4-hydroxy-2-none under oxidative stress conditions [279]. However, recent studies have shown that FSP1 inhibited ferroptosis by capturing lipid peroxide radicals on the membrane via coenzyme Q10 [280]. Second, the metal itself seems to have the ability to inhibit certain key enzymes in death pathways; for example, copper can cause the downregulation of other forms of cell death except cuproptosis, as mentioned above [146, 159]. Third, various types of cell death, such as ferroptosis, can be rapidly induced in a cascade among adjacent cells, which may be mediated by cell-cell contact. Gaps and tight junctions diffuse specific cytokines from dead cells to other cells and induce the corresponding cell death in a specific range of cells [281, 282]. Nevertheless, evidence is rare, and identical proteins may play roles in promoting different cell death processes. ELAV-like protein 1 (ELAVL1), encoding the RNA-binding protein HuR, has been shown to induce pyroptosis in cardiomyocytes under hyperglycemic conditions, and recent data revealed that upregulated ELAVL1 induced classical ferroptosis events [283, 284].

How the specific pathway is activated needs to be clarified to deepen the understanding of the mechanism underlying various death modalities. Similarly, the function of mitochondria in cell death deserves in-depth study. In addition to ROS formation and metal ion release, attention should be paid to the potential role of mitochondria themselves. Due to the involvement of the TCA cycle, the relationship between mitochondria and the cuproptosis pathway appears to be unquestionable, but researchers have different opinions on the role of mitochondria in ferroptosis. It is believed that ferroptosis is induced by lipid peroxidation outside mitochondria, and there is a situation in which mitochondria and mtDNA in cells are depleted but ferroptosis still occurs [6, 285, 286]. Although mitochondria do not play important roles in RSL3-induced ferroptosis, the evidence suggests that the mitochondrial ETC is indispensable for erastin-induced or cystine deprivation-mediated ferroptosis. Moreover, GPX4 in mitochondria exerts a more significant effect on cell resistance to ferroptosis than that exerted by GPX4 in the cytoplasm, and mitochondrial events seem to be final steps in the determination of whether cells undergo ferroptosis [287, 288]. What potential role do mitochondria play in the pathogenesis of ferroptosis? The answer to this question is of great importance to elucidate the mechanisms underlying mitochondrial quality control in ferroptosis. There may be two different pathways, a mitochondrial-dependent and mitochondrial-independent pathways. Through various pathways, “maintenance” based on mitochondrial dynamics and protein quality control and “clearance” represented by mitocytosis and mitophagy may lead to different outcomes.

Mitochondria are not isolated organelles; when the protein synthesis and transport to organelles are hindered, the function of mitochondria is affected. Imbalances in the homeostasis of metal ions, such as calcium, can influence mitochondrial function and induce cell death via ER stress. Various mechanisms underlying mitochondrial protein quality control depend on the participation of ERAD and UPS to maintain mitochondrial function as the first line of defense, and mitochondrial fusion and fission rely on the ER and lysosomes. The pathway of ferritinophagy, another autophagy modality directly affected by intracellular iron ion concentration, engages in crosstalk with the mitophagy pathway. Inhibiting the glucose flux sensing modification O-GlcNAcylation increased the rates of mitophagy and ferritinophagy, both of which released a large amount of free iron and jointly induced ferroptosis [116]. Notably, in ferroptosis, an increase in ferritinophagy activation and a decrease in mitophagy activation were noted, despite the continuous elevation in intracellular iron content, mitochondrial quantity, and ROS [289]. In addition, lipophagy, in which lipid droplets are eliminated, and clockophagy, which is based on the circadian rhythm protein ARNTL, play potential roles in ferroptosis and are associated to some degree with mitophagy [290, 291]. Although research into autophagy has been ongoing for a considerable period, we still know little about it, and the specific connections among different types of autophagy are unclear. Different types of selective autophagy depend to a certain extent on identical proteins and organelles, and lysosomes are required for the final step, which is substrate degradation. Do different autophagy pathways compete for shared resources to simultaneously interfere with each other?

The regulatory effect of metal ions on mitochondrial quality control may also be involved in cell death. Metal-induced oxidative stress caused by redox imbalances is a direct factor in the activation of mitochondrial quality control. ROS produced by the Fenton reaction promote mitochondrial hyperfusion or activate the mitochondrial fission-autophagy degradation pathway. In addition, iron and other metal ions are cofactors of many proteins. Many key proteins that regulate mitochondrial function and mitochondrial quality control, including the mitochondrial OXPHOS complex, require the participation of iron, copper and other metal elements. The Fe-S cluster is widely involved in a variety of physiological processes, and normal biogenesis of this factor is essential for life. Iron deficiency-induce Fe-S synthesis disorders affect the voltage-dependent anion channel in the mitochondrial membrane, interferes with the overall stability of mitochondria, and induces apoptosis [292]. Copper ions are involved in autophagy and are essential for the activity of autophagy kinases such as MEK1/2 and ULK1/2. Recent studies have shown that MIRO1, which induces mitochondrial membrane protrusions in MDVs, is regulated by Fe^2+^ and Ca^2+^, which provides new ideas for research in related fields [293]. Unfortunately, there is a gap in the study of the interactions between mitochondrial quality control and metal ions, the effect of metal ions on mitochondrial function has rarely been studied, and most of the relevant studies have focus only on mitochondrial dynamics and oxidative stress. There is a long way to go to apply related theories to clinical trials and human therapy.

## Conclusions

Abnormal metal ion metabolism can lead to various types of cell death. Mitochondria are centers of energy metabolism and material transport and play key roles in regulating metal ion-induced cell death. Maintaining homeostasis through mitochondrial quality control is essential for antagonizing metal ion-induced cell death. However, altering mitochondrial quality control may also promote cell death, making mitochondrial quality control a double-edged sword. In various diseases, there is a contradictory relationship between mitochondrial quality control and metal-induced cell death, and these processes have a delicate balance. For example, in neurodegenerative diseases, infections and other diseases, physiological levels of mitophagy can protect cells from pathogenic factors, but long-term uncontrolled mitochondrial quality control can exacerbate disease-related damage. In tumors, the balance between mitochondrial quality control and metal-induced cell death must be disrupted to promote tumor cell death. In fact, there are many potential factors that can modulate this balance, and it has broad application prospects in clinical practice. Future research can be expanded in related fields to provide more results and verify our hypotheses, thereby opening new paths to treat diseases.

## Data Availability

The datasets used and analyzed during the current study are available from corresponding author on reasonable request.
